# Merging artificial intelligence into cancer nursing care: Current applications, challenges, and opportunities

**DOI:** 10.1016/j.apjon.2025.100849

**Published:** 2025-12-30

**Authors:** Mian Wang, Hammoda Abu-Odah, Yingchun Zeng

**Affiliations:** a School of Nursing, The Hong Kong Polytechnic University, Hong Kong SAR, China; b Alice Lee Centre for Nursing Studies, Yong Loo Lin School of Medicine, National University of Singapore, Singapore

Cancer care is a complex, multidisciplinary field that has seen remarkable advancements in recent years. In 2022, nearly 20 million new cancer cases were diagnosed worldwide, resulting in approximately 9.7 million deaths.^1^ Notably, about half of these cases and deaths occurred in the Asia–Pacific region, highlighting the disproportionate cancer burden faced by this area compared to other regions.^1^ Projections indicate that by 2050, the global incidence of new cancer cases will surge to 35 million, further increasing pressure on health care systems.^1^ In response to this growing challenge, artificial intelligence (AI) has emerged as a transformative force, offering innovative solutions to enhance the entire cancer care continuum.^2^

## AI in cancer screening and early detection

Advanced deep learning models have significantly improved image analysis for cancer screening, enhancing diagnostic performance. A prospective multicenter study in South Korea demonstrated that AI-based computer-aided detection systems substantially improved breast cancer detection rates among radiologists, particularly for early-stage and small tumors.^3^ Sharing cancer screening tasks between AI and clinical experts is expected to reduce costs by up to 30% compared to relying solely on clinical experts.^4^ This progress is driving a growing trend toward integrating AI technologies into routine clinical workflows to improve early cancer detection and expand service capacity.

AI also enables the development of efficient and widely accessible cancer screening tools that can supplement or replace expensive traditional screening equipment. For example, Thermalytix is a portable device that combines advanced machine learning algorithms with breast thermography to detect subtle temperature variations and provide quantitative breast cancer risk assessments without radiation or compression.^5^ It is user-friendly and patient-friendly, allowing community health workers with limited training to operate it. In a large-scale study in India, Thermalytix screened over 15,000 women across diverse regions, achieving a cancer detection rate three times higher than traditional clinical breast examinations, along with timely follow-up and treatment initiation.^6^ This real-world evidence highlights the potential of AI tools to expand cancer screening coverage and enhance early detection, especially in resource-limited settings. These developments align with global health recommendations for scalable and resource-appropriate screening strategies.

Another promising trend in AI applications in cancer care is the use of advanced machine learning models to enable early cancer detection in primary care settings. Early diagnosis is crucial for improving survival rates, yet many cancers, such as lung cancer, are often detected late due to nonspecific symptoms and complex clinical presentations. Recent advances in AI, particularly transformer-based deep learning models, have shown great potential in analyzing large-scale data from widely accessible electronic health records to uncover subtle temporal patterns and clinical pathways that precede diagnosis. For example, the MedAlbert model, trained on over three million primary care records, achieved high accuracy in predicting lung cancer years before clinical diagnosis.^7^ By capturing sequential relationships among symptoms, diagnoses, and investigations, it outperformed traditional risk tools and enabled precise identification of patients at risk.^7^ Its interpretability through cluster analysis provides insights into distinct patient subgroups and clinical trajectories, enhancing clinical trust and applicability.^7^

## AI in cancer treatment planning and management

Beyond diagnosis, AI is increasingly integrated into cancer treatment planning.^8^ AI systems based on computer vision and semantic segmentation algorithms assist surgeons in identifying dissectible planes^9^ and small metastatic lesions,^10^ which optimizing tumor staging and treatment decisions. These technologies also help evaluate surgical skill performance, which is crucial for ensuring patient safety, promoting quality improvement, and enhancing surgical education.^11^ Machine learning models have also been developed to predict the risk of prolonged postoperative hospital stays^12^ and survival^13^ in patients with cancer, helping identify high-risk individuals and facilitating optimized health care resource allocation.

In radiation therapy, AI has been shown to enable precise targeting, dose control, and workflow planning.^14^ For patients undergoing systemic treatments such as chemotherapy and immunotherapy, AI applications aid in predicting post-treatment survival outcomes^13^^,^^15^ and alert clinicians to treatment-related toxicity.^16^ These advancements reflect a growing trend of integrating AI into cancer treatment to enhance precision, optimize therapeutic decisions, and improve patient safety and outcomes.

## AI applications in cancer nursing

While AI research in cancer care was initially driven largely by medical and surgical oncologists focusing on improving detection accuracy and therapeutic decision-making, recent developments have seen notable expansion led by experts in nursing, rehabilitation sciences, and psycho-social oncology. These multidisciplinary efforts broaden the impact of AI, extending benefits to patients with cancer during survivorship.

A key focus is the monitoring and prediction of treatment-related adverse events, which significantly affect patient quality of life and clinical outcomes. Zeng and colleagues developed a self-administered immersive virtual reality (VR) tool that evaluates cognitive domains such as attention, memory, processing speed, and executive function in an engaging, real-world simulated environment.^17^ The study demonstrated that this VR assessment correlates strongly with traditional neurocognitive tests, is well accepted by patients, and causes minimal discomfort.^17^ This tool offers nurses a practical, ecologically valid method to assess cognitive function in clinical settings, potentially enabling earlier identification of cognitive decline and timely intervention.

AI-powered predictive models can also help nurses identify patients at risk for other complications. For example, Wu and colleagues used machine learning models to predict lymphedema in Chinese women with breast cancer.^18^ The models were accurate and identified lifestyle factors such as exercise and diet that nurses could target with education and support. However, many patients still do not receive personalized education, which highlights the need for AI tools to assist nurses in providing precision care.

AI-based algorithms have further demonstrated advantages over the traditional Khorana score in predicting venous thromboembolism in patients with cancer.^19^^,^^20^ These algorithms can help nurses identify high-risk patients early, enabling timely thromboprophylaxis and personalized care to improve outcomes.^19^^,^^20^ Yet, current algorithm development is primarily based on retrospective cohort data, resulting in suboptimal model calibration.^19^^,^^20^ Future refinement and validation in larger prospective cohorts will be essential to develop practical AI models suitable for routine clinical nursing practice.

## AI in cancer rehabilitation

As patients with cancer live longer due to advances in cancer control and treatment, rehabilitation have become essential components of comprehensive cancer care. Health professionals are increasingly adopting AI technologies to tailor rehabilitation programs for patients with cancer.^21^

Li and colleagues’ randomized controlled trial demonstrates the promising role of AI-driven digital therapeutics in cardio-oncology rehabilitation, where an AI-tailored exercise prescription delivered via a mobile app and wearable devices significantly improved cardiopulmonary fitness and quality of life in patients with early-stage non-small cell lung cancer.^22^ Complementing this, Qi and colleagues provide evidence for the feasibility and benefits of an integrated rehabilitation model based on mobile health and virtual reality that combines exercise, nutrition, and psychological support for patients with cancer.^23^ This multidisciplinary model, delivered by a collaborative team of oncologists, dietitians, psychotherapists, and nurse specialists, leverages digital health technologies such as mobile health applications for real-time monitoring and virtual reality interventions for psychological well-being to enhance patient engagement, adherence, and multidimensional recovery.

## AI in end-of-life cancer care

End-of-life care for patients with cancer has long been challenged by diagnostic and prognostic uncertainties, which often hinder timely decision-making and appropriate care planning. Recently, AI has begun to make inroads into this complex area, offering promising opportunities to enhance prognostication and personalize treatment strategies. For instance, He and colleagues developed an interpretable machine learning model using the CatBoost algorithm on a large cohort of 1893 critically ill patients with cancer and delirium.^24^ Their model accurately predicted 28-day mortality and identified key clinical predictors such as Glasgow Coma Scale scores and medication use.^24^ Importantly, the model's interpretability, achieved through SHapley Additive exPlanations analysis, provides clinicians with actionable insights for early risk stratification and targeted interventions.^24^

Beyond predictive capabilities, AI also plays a vital role in improving the delivery of quality end-of-life care. Manz and colleagues’ randomized clinical trial demonstrated that a machine learning algorithm predicting six-month mortality, combined with behavioral nudges directed at oncology clinicians, significantly increased the occurrence of serious illness conversations and reduced the use of systemic therapy near the end of life.^25^ This intervention underscores the potential of AI to facilitate timely, patient-centered communication and shared decision-making, thereby enhancing the overall quality of end-of-life care.

## Understanding patient experience through AI

Patient experience is a critical factor influencing treatment adherence, satisfaction, and outcomes throughout the cancer care journey. Understanding the emotional, informational, and social needs of patients is essential for truly patient-centered care. Traditional qualitative methods like interviews and surveys face limitations such as small sample sizes, time demands, and potential biases.

Consequently, AI applications are increasingly used to provide scalable, data-driven insights into patient experiences. A recent study by Voigt and colleagues exemplifies this trend by applying machine learning topic modeling to analyze over 200,000 posts from colorectal cancer forums.^26^ This approach uncovered diverse patient concerns, including treatment understanding, emotional challenges at home, and daily life management, offering a comprehensive view of the patient journey rarely achievable with conventional methods.^26^ The study also shows how AI can efficiently process large volumes of unstructured data to inform remote monitoring and personalized care.^26^ By integrating AI analysis with clinical expertise, such methods enhance understanding of patient needs and support the development of responsive, value-based cancer care.^26^

## Future directions and integration

Looking ahead, AI in cancer care is expected to become increasingly multidisciplinary and integrated. Advances in multimodal data fusion, which combines imaging, clinical records, patient-generated data, and genomics, will enable the development of more comprehensive and precise predictive models. Personalized medicine stands to benefit greatly from the ability of AI to tailor interventions to individual patient profiles.

As cancer survival rates improve, more patients are transitioning from hospital-based treatment to community care. This shift creates a need for integrated care models that connect cancer care with primary care services. The onco-primary care model has been proposed to address the complex needs of patients with cancer in community settings by fostering coordinated management between cancer specialists and primary care generalists.^27^ One major obstacle to adopting this model is ensuring seamless information exchange across different care environments.^27^ AI technologies, including natural language processing and interoperable data platforms, offer promising solutions to overcome these challenges. Such innovations will be essential to meeting the evolving demands of cancer care in the community.

## Challenges and ethical considerations

Despite these advances, significant challenges remain. Rigorous clinical validation is essential to ensure the safety and effectiveness of AI tools. Ethical considerations, including data privacy, algorithmic bias, and informed consent, must be carefully addressed. Moreover, fostering collaboration among oncologists, nurses, rehabilitation specialists, psychosocial professionals, data scientists, and patients is crucial to fully realize the transformative potential of AI in cancer care.

An important concern is that current AI models used for medical imaging analysis may inadvertently predict individuals’ self-reported race, a capability that is often difficult to detect and isolate.^28^ This phenomenon could contribute to racial disparities in health outcomes.^28^ Future research should explore the specific impact and underlying mechanisms of this issue on cancer care and patient outcomes, providing valuable insights to guide scrutiny and control measures in clinical practice.

## Conclusions

AI is transforming cancer nursing care by seamlessly merging into every stage of the patient journey, from supporting early detection and diagnosis to facilitating personalized treatment, survivorship management, and compassionate end-of-life support. The expanding role of oncology nurses in collaboration with interdisciplinary teams highlights the multidisciplinary essence of cancer care, enriching AI applications tailored to nursing workflows. By navigating current applications alongside inherent challenges—such as integration barriers and ethical considerations—while seizing opportunities for innovation and rigorous evaluation, AI holds immense promise for enhancing patient outcomes, reducing nurse workloads, and elevating care experiences globally.

## CRediT authorship contribution statement

**Mian Wang**, **Hammoda Abu-Odah**: Conceptualization, Writing – Original Draft; **Yingchun Zeng**: Supervision, Writing – Review & Editing. All authors have read and approved the final manuscript.

## Ethics statement

Not required.

## Data availability statement

Data availability is not applicable to this article as no new data were created or analyzed in this study.

## Declaration of generative AI and AI-assisted technologies in the writing process

No AI tools/services were used during the preparation of this work.

## Funding

This study received no external funding.

## Declaration of competing interest

The authors declare no conflict of interest. The corresponding author, Dr. Yingchun Zeng, serves as a member of the editorial board of the *Asia–Pacific Journal of Oncology Nursing*. The article has undergone the journal's standard publication procedures.

## References

[1] Bray F., Laversanne M., Sung H. (2024). Global cancer statistics 2022: GLOBOCAN estimates of incidence and mortality worldwide for 36 cancers in 185 countries. CA Cancer J Clin.

[2] Riaz I.B., Khan M.A., Osterman T.J. (2025). Artificial intelligence across the cancer care continuum. Cancer.

[3] Chang Y.W., Ryu J.K., An J.K. (2025). Artificial intelligence for breast cancer screening in mammography (AI-STREAM): preliminary analysis of a prospective multicenter cohort study. Nat Commun.

[4] Ahsen M.E., Ayvaci M.U.S., Mookerjee R. (2025). Economics of AI and human task sharing for decision making in screening mammography. Nat Commun.

[5] Niramai Health Analytix Thermalytix: AI-powered breast cancer screening test. https://niramai.com/about/thermalytix/.

[6] Adapa K., Gupta A., Singh S. (2025). A real world evaluation of an innovative artificial intelligence tool for population-level breast cancer screening. npj Digit Med.

[7] Wang L., Yin Y., Glampson B. (2024). Transformer-based deep learning model for the diagnosis of suspected lung cancer in primary care based on electronic health record data. EBioMedicine.

[8] Nashwan A.J., Bani Hani S. (2023). Enhancing oncology nursing care planning for patients with cancer through harnessing large language models. Asia Pac J Oncol Nurs.

[9] Kumazu Y., Kobayashi N., Senya S. (2025). AI-based visualization of loose connective tissue as a dissectable layer in gastrointestinal surgery. Sci Rep.

[10] Chen H., Gou L., Fang Z. (2025). Artificial intelligence assisted real-time recognition of intra-abdominal metastasis during laparoscopic gastric cancer surgery. npj Digit Med.

[11] Matsumoto S., Kawahira H., Fukata K. (2024). Laparoscopic distal gastrectomy skill evaluation from video: a new artificial intelligence-based instrument identification system. Sci Rep.

[12] Zhang G., Liu X., Hu Y. (2024). Development and comparison of machine-learning models for predicting prolonged postoperative length of stay in lung cancer patients following video-assisted thoracoscopic surgery. Asia Pac J Oncol Nurs.

[13] Yuan Y., Zhang G., Gu Y. (2025). Artificial intelligence-assisted machine learning models for predicting lung cancer survival. Asia Pac J Oncol Nurs.

[14] Yu L., Ni Q., Wang B. (2025). Multicenter study on the versatility and adoption of AI-driven automated radiotherapy planning across cancer types. Nat Commun.

[15] Lippenszky L., Mittendorf K.F., Kiss Z. (2024). Prediction of effectiveness and toxicities of immune checkpoint inhibitors using real-world patient data. JCO Clin Cancer Inform.

[16] Oikonomou E.K., Sangha V., Dhingra L.S. (2025). Artificial intelligence-enhanced risk stratification of cancer therapeutics-related cardiac dysfunction using electrocardiographic images. Circ Cardiovasc Qual Outcomes.

[17] Zeng Y., Zeng L., Cheng A.S.K. (2022). The use of immersive virtual reality for cancer-related cognitive impairment assessment and rehabilitation: a clinical feasibility study. Asia Pac J Oncol Nurs.

[18] Wu X., Guan Q., Cheng A.S.K. (2022). Comparison of machine learning models for predicting the risk of breast cancer-related lymphedema in Chinese women. Asia Pac J Oncol Nurs.

[19] Meng L., Wei T., Fan R. (2022). Development and validation of a machine learning model to predict venous thromboembolism among hospitalized cancer patients. Asia Pac J Oncol Nurs.

[20] Jin S., Qin D., Wang C. (2025). Development, validation, and clinical utility of risk prediction models for cancer-associated venous thromboembolism: a retrospective and prospective cohort study. Asia Pac J Oncol Nurs.

[21] Zeng Y., Tang Q., Chen S., Chen X. (2023). Integration of a large language model with augmentative and alternative communication tool for oncological aphasia rehabilitation. Asia Pac J Oncol Nurs.

[22] Li G., Zhou X., Deng J. (2025). Digital therapeutics-based cardio-oncology rehabilitation for lung cancer survivors: randomized controlled trial. JMIR mHealth uHealth.

[23] Qi Y., Wang M., Xue Y. (2024). Feasibility of an exercise-nutrition-psychology integrated rehabilitation model based on mobile health and virtual reality for cancer patients: a single-center, single-arm, prospective phase II study. BMC Palliat Care.

[24] He Y., Liu N., Hao S., Xu M., Zeng Y. (2025). Interpretable machine learning-based prediction of mortality in critical cancer patients with delirium: a retrospective cohort study. Asia Pac J Oncol Nurs.

[25] Manz C.R., Zhang Y., Chen K. (2023). Long-term effect of machine learning-triggered behavioral nudges on serious illness conversations and end-of-life outcomes among patients with cancer: a randomized clinical trial. JAMA Oncol.

[26] Voigt K., Sun Y., Patandin A. (2025). A machine learning approach using topic modeling to identify and assess experiences of patients with colorectal cancer: explorative study. JMIR Cancer.

[27] Oeffinger K.C., Corbett C., Shah K.P. (2025). Oncology, primary care, and survivorship: time for onco-primary care?. JCO Oncol Pract.

[28] Gichoya J.W., Banerjee I., Bhimireddy A.R. (2022). AI recognition of patient race in medical imaging: a modelling study. Lancet Digit Health.

